# A novel innate immunity‐mediated senescence mechanism regulated by cGAS–STING–IRF3–pRB

**DOI:** 10.1002/mco2.70072

**Published:** 2025-01-15

**Authors:** Linbin Zhou, Jiahui Li, Wai Kit Chu

**Affiliations:** ^1^ Department of Ophthalmology & Visual Sciences The Chinese University of Hong Kong Hong Kong SAR China; ^2^ Hong Kong Hub of Paediatric Excellence The Chinese University of Hong Kong Hong Kong SAR China

1

In a study recently published in *Science Advances*, Wu et al.^1^ demonstrated that the retinoblastoma protein (pRB) engages the cyclic GMP–AMP synthase (cGAS)–stimulator of interferon genes (STING) cascade via interacting with interferon regulatory factor 3 (IRF3) to initiate senescence in hepatic stellate cells, which hampers liver fibrosis. These intriguing findings are important for developing novel therapeutic strategies for inducing senescence via manipulating the cGAS–STING–IRF3–pRB cascade.

The cyclic cGAS–STING pathway functions as an innate immune defense primed by cytosolic DNA. Upon sensing cytosolic DNA, cGAS–STING recruits and activates the inhibitor of NF‐κB kinase (IKK) and TANK‐binding kinase 1 (TBK1). The activated IKK and TBK1 activate nuclear factor kappa‐B (NF‐κB) and IRF3, respectively, which, in turn, enter the nucleus and induce the expression of multiple cytokines.^2^ Hitherto, the cGAS–STING signaling has been demonstrated to play crucial roles in various biological processes, ranging from autophagy and cellular condensation to cell death.^2^ Deregulation of the cGAS–STING cascade frequently results in autoimmune, infectious and degenerative diseases, and cancers.^2^ A crucial role of the cGAS–STING cascade in driving senescence has recently emerged,^2^ yet the underlying mechanisms of cGAS–STING‐induced senescence need more investigations.

pRB functions as a negative regulator of the cell cycle by controlling the G1 to S phase transition, where pRB is inactivated via cyclin‐dependent kinases (CDK)‐mediated hyperphosphorylation, allowing E2F to release from the pRB–E2F complex.^3^ E2F can then induce the expression of genes required for the G1 to S phase transition.^3^ As such, pRB has long been viewed as one of the central regulators of senescence, a cellular state with irreversible cell cycle arrests. Recent findings from Wu and colleagues demonstrated that pRB is involved in the cGAS–STING‐induced senescence under DNA damage conditions, where phosphorylated IRF3 outcompetes CDK4/6 in interacting with pRB to maintain pRB in a hypophosphorylation state, which promotes pRB–E2F binding and hampers E2F‐induced gene expression (Figure 1).^1^

**FIGURE 1:**
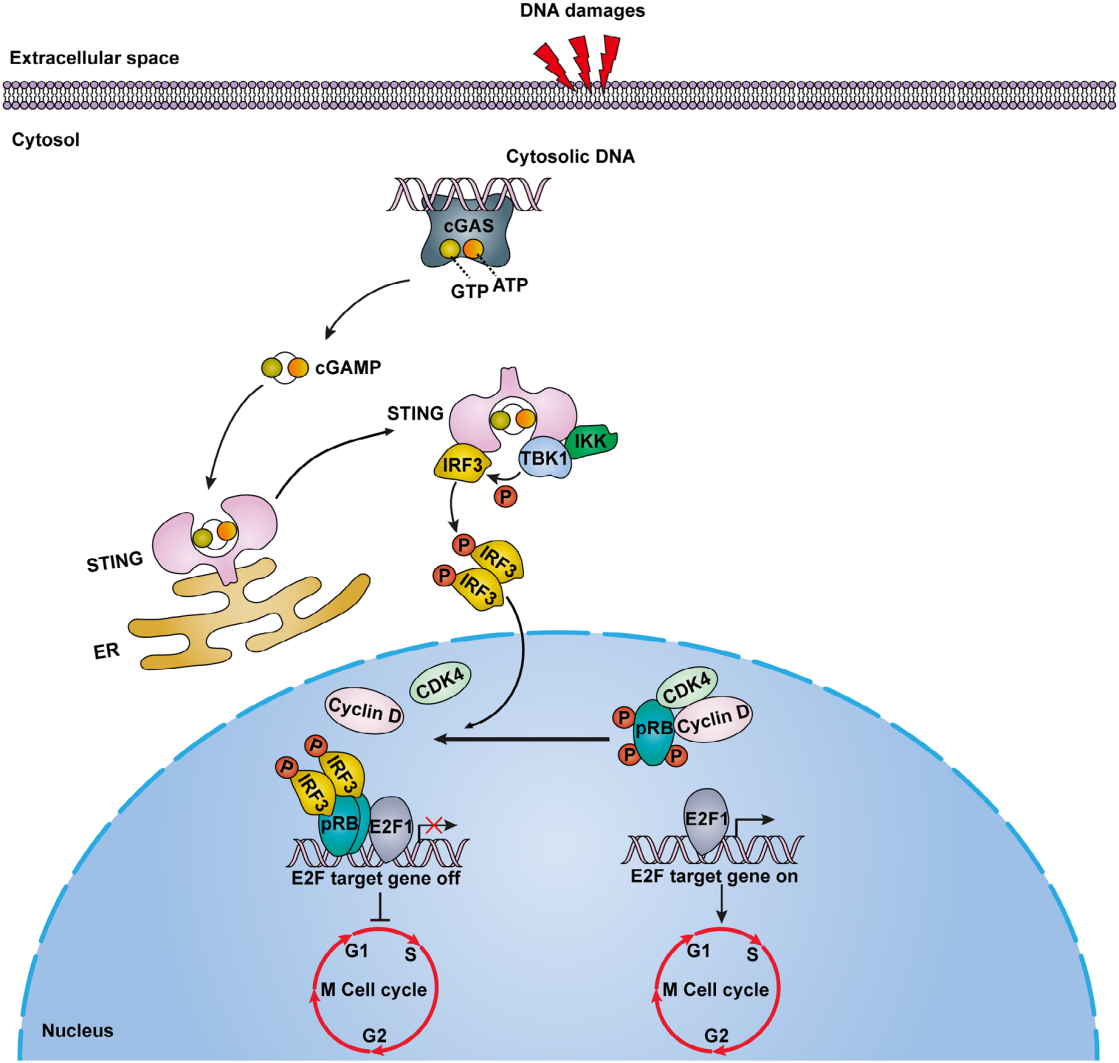
The retinoblastoma protein (pRB) is involved in the cyclic guanosine monophosphate–adenosine monophosphate (GMP–AMP) synthase (cGAS)–stimulator of interferon genes (STING) signaling to initiate cell cycle arrests under DNA damages. Under DNA damages, aberrant cytosolic DNA is sensed by cGAS, which catalyzes guanosine triphosphate (GTP) and adenosine triphosphate (ATP) to produce a secondary messenger cyclic GMP–AMP (cGAMP). The cGAMP associates with and activates endoplasmic reticulum (ER)‐associated transmembrane protein STING. STING subsequently recruits and activates the inhibitor of NF‐κB kinase (IKK) and TANK‐binding kinase 1 (TBK1). The activated TBK1 in turn phosphorylates interferon regulatory factor 3 (IRF3). The phosphorylated IRF3 forms dimers and translocates into the nucleus, where IRF3 disrupts the pRB–cyclin‐dependent kinase 4 (CDK4)–cyclin D complex via binding to pRB, leading to a hypophosphorylated state of pRB and an enhanced E2F transcription factor 1 (E2F1)–pRB binding. Consequently, E2F1 target gene transcription is suppressed, and the G1 to S phase transition of the cell cycle is blocked.

Wu and colleagues observed cGAS–STING signaling activation and senescence after DNA damage induction in various cells. IRF3 ablation suppressed the DNA damage‐induced senescence in these cells. Furthermore, a marked decrease in pRB phosphorylation levels was observed in DNA damage‐induced senescent cells, while IRF3 ablation reversed it. Conversely, in IRF3‐ablated cells, reintroducing IRF3 restored pRB hypophosphorylation under DNA damage‐induced senescence. Importantly, pRB was further identified as a prominent IRF3‐binding protein. Notably, among the components of the cGAS–STING signaling, only IRF3 specifically interacted with pRB. Further domain mapping assays revealed that the interferon‐activating domain of IRF3 and the pocket B domain of pRB were essential for their binding.^1^

In cell cycle regulation, CDK‐mediated pRB phosphorylation is pivotal in modulating pRB interaction with E2Fs and other transcription factors.^3^ Wu and colleagues found that IRF3–pRB interaction selectively inhibited CDK4‐mediated pRB phosphorylation in the presence of cyclin D1. Moreover, IRF3, pRB, and E2F1 formed a tripartite complex in DNA damage‐induced senescent cells.^1^ Dephosphorylated pRB can sequester E2F1 and result in cell proliferation arrest.^3^ STING expression conferred, but IRF3 knockout reversed, cell proliferation arrests.^1^ Besides, they further found that the cGAS–STING–IRF3–pRB axis promoted senescence and impeded liver fibrosis, while hepatic stellate cell‐specific *Irf3* knockout mitigated senescence yet exacerbated liver fibrosis in liver fibrosis mouse models,^1^ which underscores an important implication of the cGAS–STING–IRF3–pRB axis‐mediated senescence in liver fibrosis.

Senescence mediated by the cGAS–STING pathway emerges to play a role in organ fibrosis. The STING–PKR‐like endoplasmic reticulum kinase (PERK)–eIF2α pathway triggered the onset of senescence and hampered lung and renal fibrosis, whereas the cell types responsible for the antifibrotic effects have not been specified.^2^ The cGAS–STING–IRF3–pRB cascade also contributed to initiating and reinforcing senescence and consequently retarding liver fibrosis.^1^ Particularly, the hepatic stellate cells, instead of hepatocytes, were identified as a critical mediator in the cGAS–STING–IRF3–pRB cascade‐mediated senescence,^1^ which provides a specific cell type for the future development of precise therapeutic strategies in liver fibrosis. Despite these inspiring findings, several important issues remain to be addressed. First, though the IRF3–pRB interaction did not affect the classical transcriptional functions of IRF3 in inducing IFN gene expression, the senescence‐associated secretory phenotype (SASP) from the senescent hepatic stellate cells may affect the functions of the neighboring hepatocytes. Second, *Irf3* activation was reported to exacerbate bile duct ligation‐induced liver fibrosis, while global *Irf3* knockout impeded liver fibrosis development.^4^ Unlike the study conducted by Wu and colleagues where hepatic stellate cells were identified as the critical cell type,^1^ this study did not specify a cell type responsible for the *Irf3* knockout effects on liver fibrosis,^4^ which limits the development of a more precise therapeutic strategy. Additionally, further studies are still required to address the contradicting results between these two studies. Third, apart from hepatic stellate cells, other cell types, such as macrophages and endothelial cells,^5^ may also be a crucial mediator in cGAS–STING–IRF3–pRB cascade‐mediated senescence in liver fibrosis. Yet, it should be noted that *p16^Ink4a+^* senescent macrophages and endothelial cells played distinct roles in liver fibrosis, with the former exacerbating and the latter mitigating liver fibrosis respectively.^5^ Therefore, future studies are warranted to investigate their possible involvement in cGAS–STING–IRF3–pRB cascade‐mediated senescence in liver fibrosis, which may help develop novel liver fibrosis interventions through precise targeting of cell type‐specific senescence.

Reversibly governing G1 to S phase transition of the cell cycle is a well‐recognized canonical function of pRB, where pRB is phosphorylated by CDK to activate E2F for cell exiting quiescence and entering proliferation phases.^3^ By contrast, pRB is also a crucial mediator in senescence, an irreversible cell cycle arrest state, through a conventional p16^Ink4a^–pRB mechanism triggered by various stresses, including aging, DNA damage, and oxidative stress. Besides permanently exiting the cell cycle, senescent cells exhibit an SASP phenotype and undergo metabolic reprogramming, distinct from quiescent cells. For the first time, pRB has been demonstrated to trigger cell cycle arrest and initiate senescence entry through a novel cGAS–STING–IRF3–pRB mechanism (Figure 1).^1^ Senescence of hepatic stellate cells mediated by the cGAS–STING–IRF3–pRB mechanism retarded liver fibrosis development.^1^ Apart from liver fibrosis, cGAS–STING–IRF3–pRB cascade‐mediated senescence could also be involved in other human diseases, such as cancers, which deserve further investigation.

To summarize, the cGAS–STING–IRF3–pRB cascade mediates cellular senescence in hepatic stellate cells to impede liver fibrosis, which enables the development of precise therapeutic strategies. Future works are warranted to investigate the potential roles of other cell types in cGAS–STING–IRF3–pRB cascade‐mediated senescence in liver fibrosis and the roles of the cGAS–STING–IRF3–pRB pathway in other human diseases, such as cancers.

## AUTHOR CONTRIBUTIONS

*Conceptualization (lead); writing—review and editing (equal); funding acquisition (lead)*: Wai Kit Chu. *Writing—original draft (equal); writing—review and editing (equal); visualization (lead)*: Linbin Zhou. *Writing—original draft (equal); writing—review and editing (equal)*: Jiahui Li. All authors have read and approved the final manuscript.

## CONFLICT OF INTEREST STATEMENT

The authors declare no conflicts of interest.

## ETHICS STATEMENT

Not applicable.

## Data Availability

Not applicable.
